# Author Correction: *O*-GlcNAcylation of MITF regulates its activity and CDK4/6 inhibitor resistance in breast cancer

**DOI:** 10.1038/s41467-026-72046-y

**Published:** 2026-04-13

**Authors:** Yi Zhang, Shuyan Zhou, Yan Kai, Ya-qin Zhang, Changmin Peng, Zhuqing Li, Muhammad Jameel mughal, Belmar Julie, Xiaoyan Zheng, Junfeng Ma, Cynthia X. Ma, Min Shen, Matthew D. Hall, Shunqiang Li, Wenge Zhu

**Affiliations:** 1 Department of Biochemistry and Molecular Medicine, GWU Cancer Center, George Washington University School of Medicine and Health Sciences, Washington, DC USA; 2 Division of Preclinical Innovation (Intramural), National Center for Advancing Translational Sciences (NCATS), National Institutes of Health, Rockville, MD USA; 3 Department of Medicine, Washington University School of Medicine in St Louis, Siteman Cancer Center, St Louis, MO USA; 4 Department of Anatomy and Cell Biology, GWU Cancer Center, George Washington University School of Medicine and Health Sciences, Washington, DC USA; 5 Department of Oncology, Lombardi Comprehensive Cancer Center, Georgetown University Medical Center, Washington, DC USA; 6 Division of Oncology, Department of Internal Medicine, Washington University School of Medicine, St. Louis, MO USA

**Keywords:** High-throughput screening, Breast cancer, Glycosylation

Correction to: *Nature Communications* 10.1038/s41467-024-49875-w, published online 03 July 2024

In the version of this article initially published, the palbociclib image in Fig. 2k was an inadvertent duplicate of the palbociclib image in Fig. 2j. Source data containing the original, uncropped images used to generate the figure are available (10.6084/m9.figshare.31356262). The figure has been updated in the HTML and PDF versions of the article.

