# Supplementary material for: O-GlcNAcylation of MITF regulates its activity and CDK4/6 inhibitor resistance in breast cancer
Source: Nat Commun. 2024 Jul 3;15:5597. doi: 10.1038/s41467-024-49875-w (PMC11222436; doi:10.1038/s41467-024-49875-w)
Supplement: Supplementary file 1 — Supplementary Information [file 41467_2024_49875_MOESM1_ESM.pdf]

O-GlcNAcylation of MITF regulates its activity and CDK4/6 inhibitor resistance in breast cancer

Supplementary Information

This file includes:

Supplementary Figure 1. qHTCS identifies MITF inhibitor ML329 that overcomes palbociclib resistance

Supplementary Figure 2. Inhibition of MITF overcomes palbociclib resistance by activating the senescence pathway in breast cancer cells

Supplementary Figure 3. OGT interacts with MITF and promotes its nuclear translocation

Supplementary Figure 4. O-GlcNAcylation of MITF at S49 is required for nuclear accumulation and resistance

Supplementary Figure 5. O-GlcNAcylation within the nuclear localization signal (NLS) promotes the interaction of MITF with importin α/β

Supplementary Figure 6. MITF is activated in response to palbociclib and elevated in tumors from palbociclib-resistant breast cancer patients

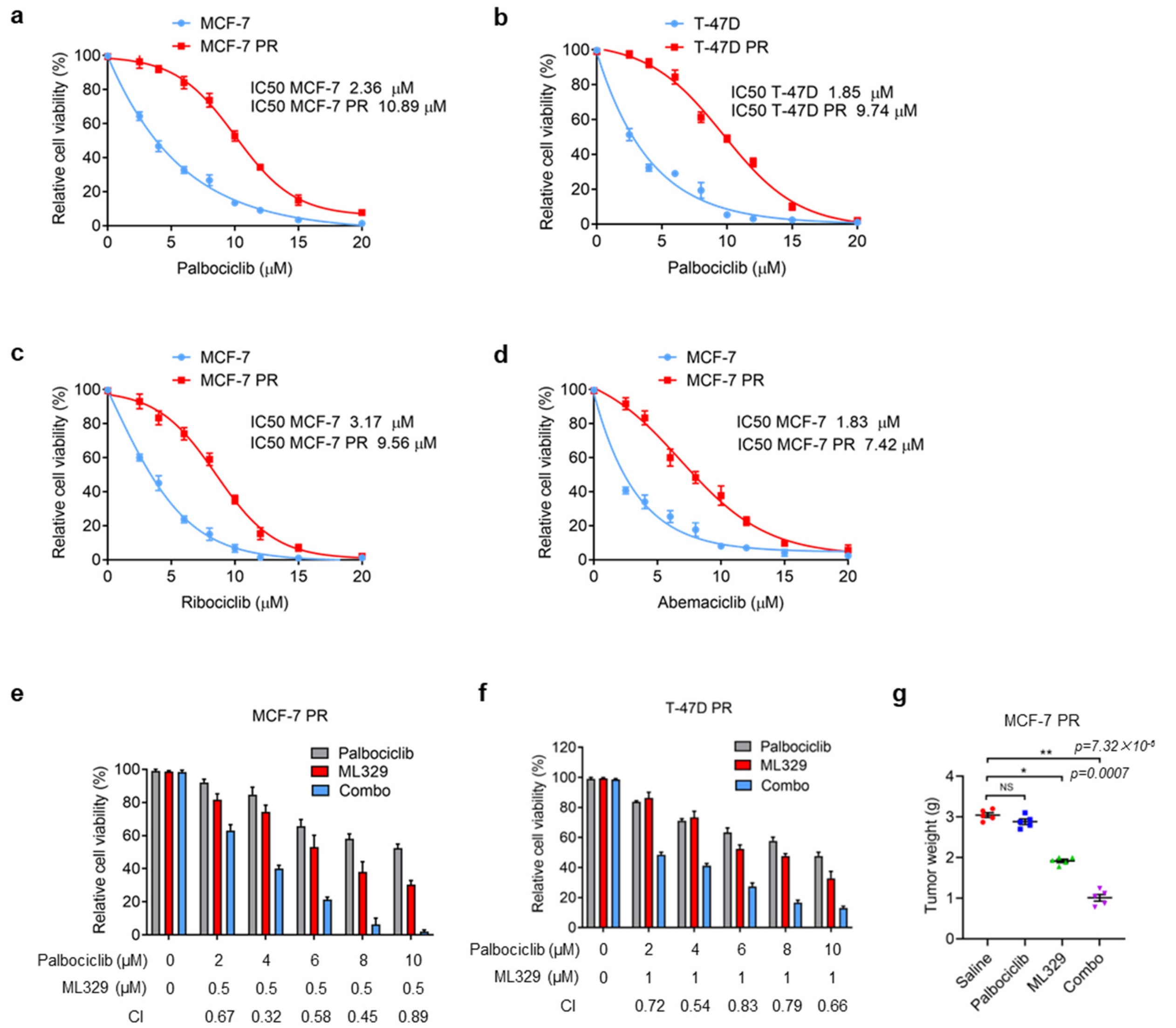

Supplementary Figure 1. qHTS identifies MITF inhibitor ML329 that overcomes palbociclib resistance

(**a-b**) Viability of MCF-7 and MCF-7 PR cells (**a**), T-47D and T-47D PR cells (**b**) treated with increasing concentrations of palbociclib for 3 days (n = 3 independent experiments). The IC_{50} was indicated. (**c-d**) Viability of MCF-7 PR cells treated with increasing concentrations of ribociclib (**c**) and abemaciclib (**d**) for 3 days (n = 3 independent experiments). The IC_{50} was indicated. (**e-f**) The synergistic effects of ML329 and palbociclib on MCF-7 PR cells (**e**) and T-47D PR cells (**f**) (n = 3 independent experiments). CI values are presented below the bars. (**g**) Tumor weight of MCF-7 PR xenografts was assessed after treatments with the following compounds: saline, palbociclib (25mg/kg), ML329 (10mg/kg), and a combination of palbociclib with ML329 for 2

weeks. n = 6 mice/group. **, $p \leq 0.01$, *, $p \leq 0.05$. All error bars are expressed as mean ± SEM. Two-tailed Student's t-tests were employed for statistical evaluation. Source data are provided as a Source Data file.

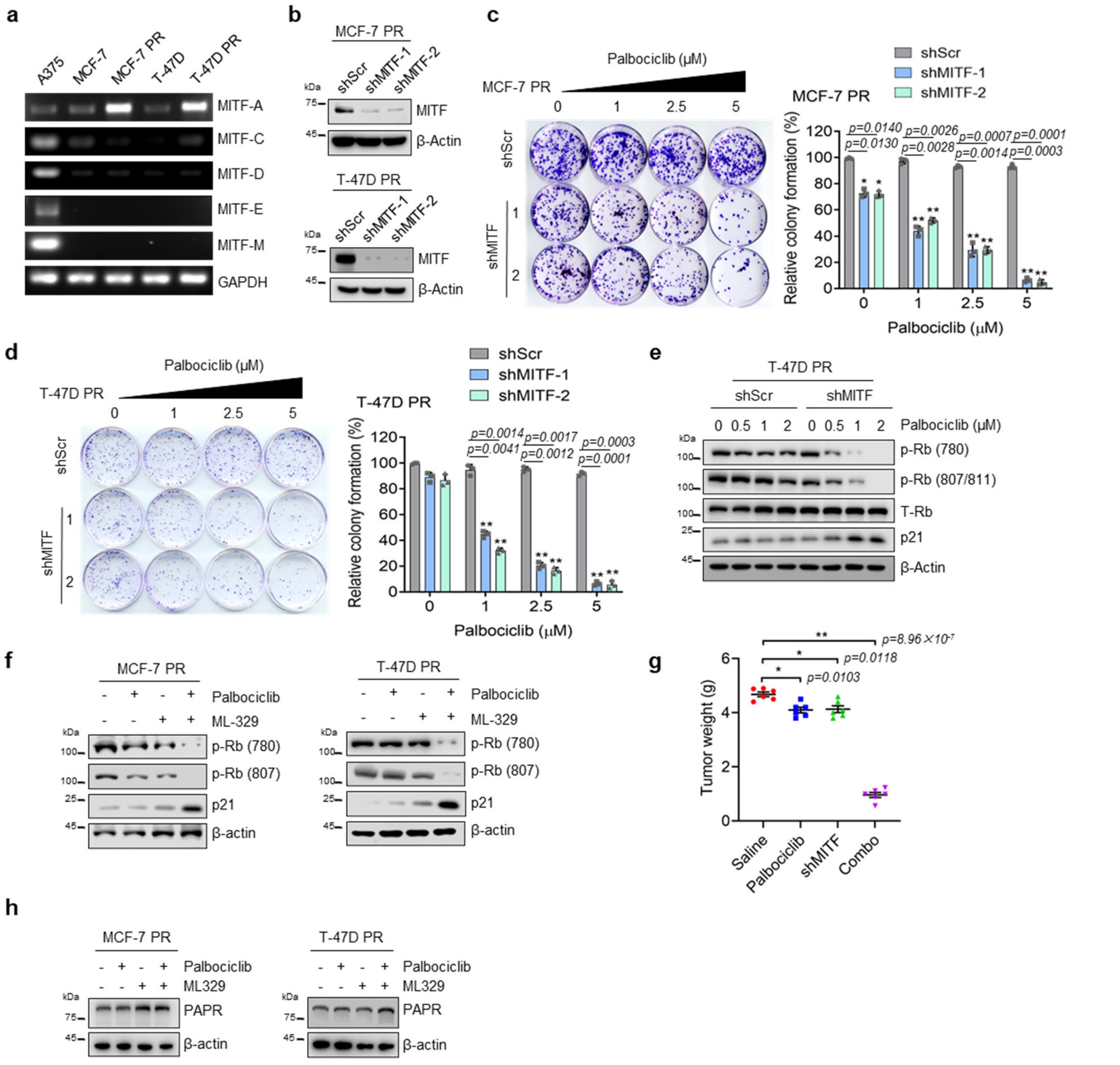

Supplementary Figure 2. Inhibition of MITF overcomes palbociclib resistance by activating the senescence pathway in breast cancer cells

(**a**) RT–PCR analysis to examine the expression levels of MITF isoforms in cells as indicated. Note, MITF-M was detected in A375 melanoma cells but not in breast cancer cells. (**b**) MCF-7 PR and T-47D PR cells treated as indicated were collected and then followed by immunoblotting for the indicated proteins. (n = 3 independent experiments). (**c-d**) Representative images of colony

formation and quantification results in MCF-7 PR cells (**c**) and T-47D PR cells (**d**) with indicated treatments (n = 3 independent experiments). (**e**) T-47D PR cells treated as indicated were collected and then followed by immunoblotting for the indicated proteins. (n = 3 independent experiments). (**f**) MCF-7 PR and T-47D PR cells treated as indicated were collected and then followed by immunoblotting for the indicated proteins. (n = 3 independent experiments). (**g**) Tumor weight of MCF-7 PR xenograft treated with saline, palbociclib (25mg/kg), ML329(5mg/kg), or a combination of both for 3 weeks. n = 6 mice/group. (**h**) MCF-7 PR and T-47D PR cells treated as indicated were collected and then immunoblotted for indicated proteins. (n = 3 independent experiments). **, $p \le 0.01$, *, $p \le 0.05$. All error bars are expressed as mean ± SEM. Two-tailed Student's t-tests were employed for statistical evaluation. Source data are provided as a Source Data file.

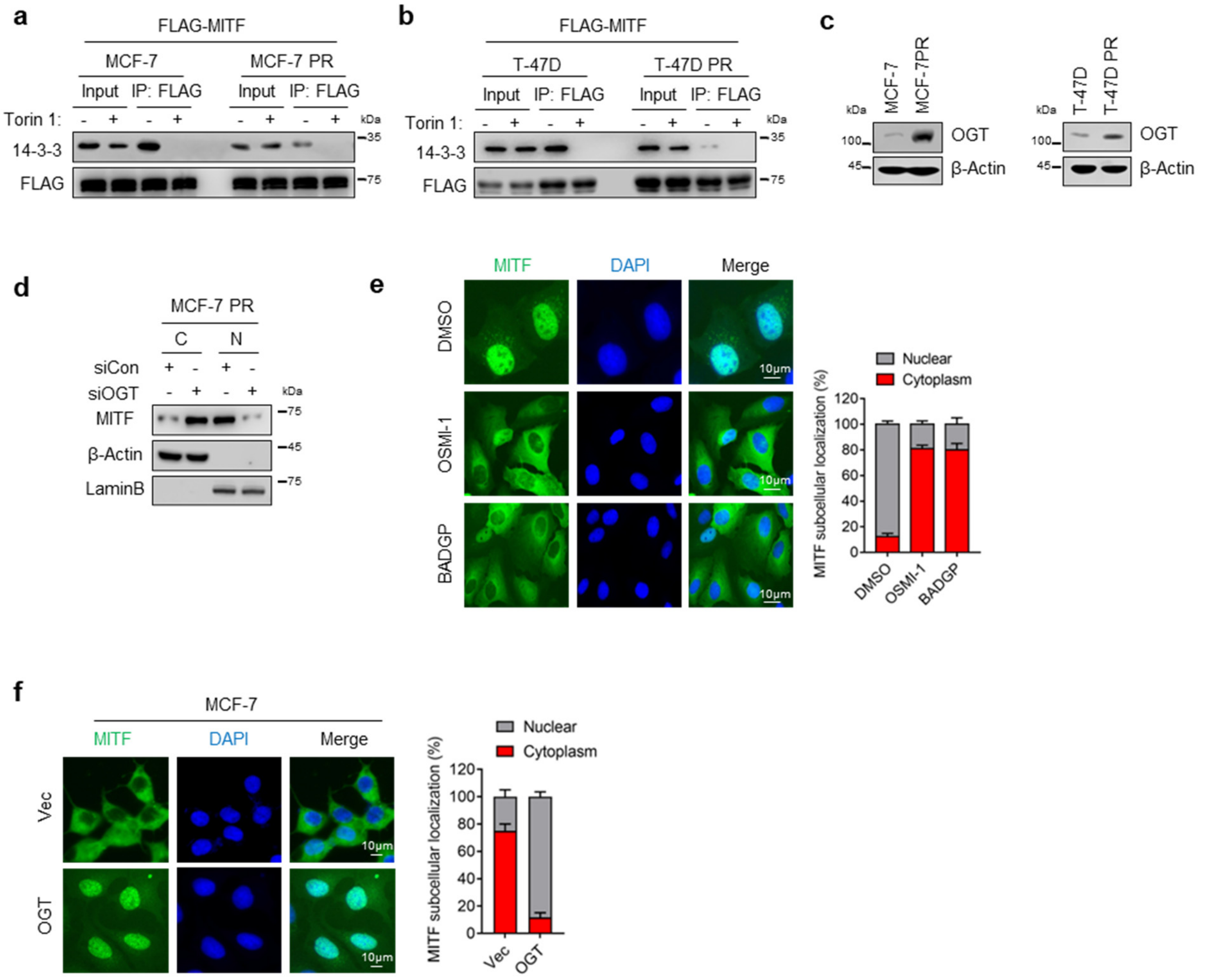

Supplementary Figure 3. OGT interacts with MITF and promotes its nuclear translocation

(**a**) MCF-7 and MCF-7 PR cells were transfected with the indicated plasmids for 48 hr before being harvested. FLAG-IPs were immunoblotted for the indicated proteins. (n = 3 independent experiments). (**b**) T-47D and T-47D PR cells were transfected with the indicated plasmids for 48 hr before being harvested. FLAG-IPs were immunoblotted for the indicated proteins. (n = 3 independent experiments). (**c**) MCF-7 & MCF-7 PR cells and T-47D & T-47D PR cells were collected and followed by immunoblotting for the indicated proteins. (n = 3 independent experiments). (**d**) MCF-7 PR cells were collected after indicated treatments, and then cytoplasmic and nuclear fractions were separated and cell lysates were subjected to immunoblotting for

indicated proteins. (n = 3 independent experiments). (**e**) MCF-7 PR cells were treated as indicated, followed by immunostaining to examine the localization of MITF (n = 3 independent experiments). The scale bar represents 10 μm. Right panel, quantification results. (**f**) MCF-7 cells were treated as indicated, and then followed by immunostaining to examine the localization of MITF (n = 3 independent experiments). The scale bar represents 10 μm. Right panel, quantification results. All error bars are expressed as mean ± SEM. Two-tailed Student's t-tests were employed for statistical evaluation. Source data are provided as a Source Data file.

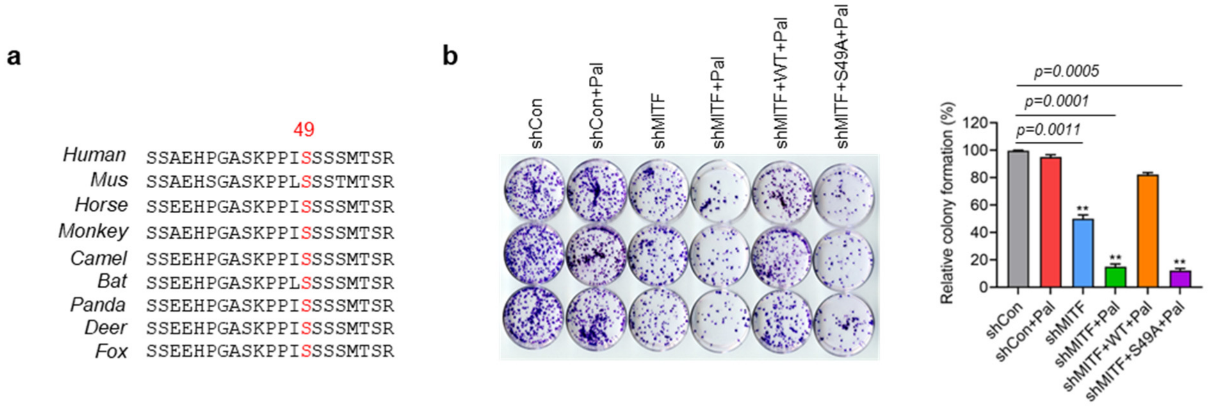

Supplementary Figure 4. O-GlcNAcylation of MITF at S49 is required for its nuclear accumulation and resistance

(**a**) MITF S49 site is conserved across various species. (**b**) Left panel, representative images of colony formation in MCF-7 PR cells with indicated treatments (n = 3 independent experiments). Right panel, quantification results. **, p≤0.01. All error bars are expressed as mean ± SEM. Two-tailed Student's t-tests were employed for statistical evaluation. Source data are provided as a Source Data file.

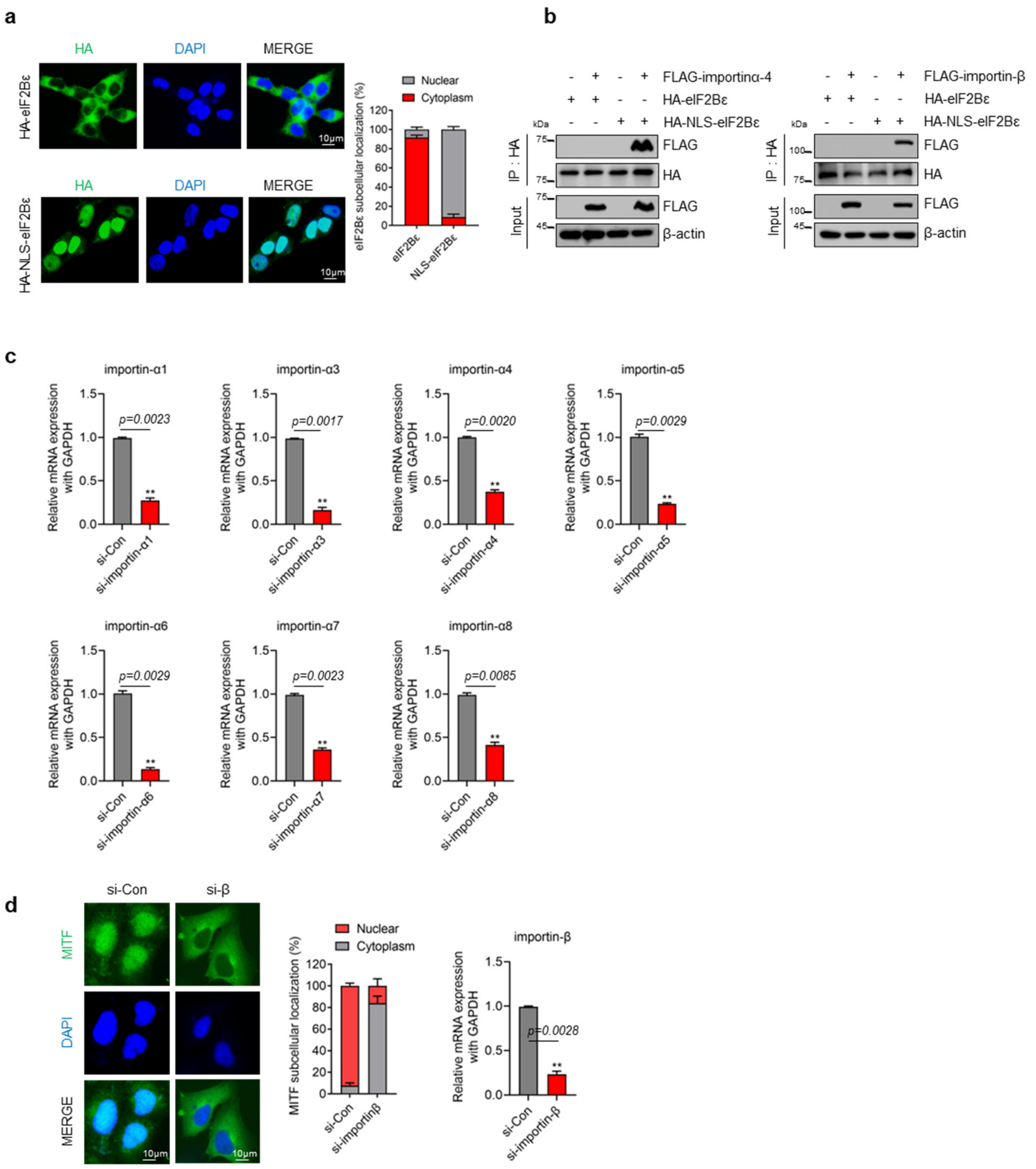

Supplementary Figure 5. O-GlcNAcylation within the nuclear localization signal (NLS) promotes the interaction of MITF with importin α/β and nuclear translocation

(**a**) pcDNA3.1-HA-eIF2Bε and pcDNA3.1-HA-NLS-eIF2Bε plasmids were transfected into MCF-7 PR cells as indicated, and the eIF2Bε cellular localization was examined by immunostaining (n

= 3 independent experiments). The scale bar represents 10 μm. (**b**) MCF-7 PR cells were transfected with indicated plasmids for 48 hr before being harvested for co-IP. HA-IPs were then immunoblotted for indicated proteins. (n = 3 independent experiments). (**c**) MCF-7 PR cells were collected after the indicated treatment and subjected to qPCR to examine the expression of indicated genes (n = 3 independent experiments). (**d**) MCF-7 PR cells were transfected with the indicated siRNAs and then followed by immunostaining to detect MITF cellular localization (n = 3 independent experiments). The scale bar represents 10 μm. Right panel, quantification results are shown on the left. **, $p \leq 0.01$. All error bars are expressed as mean ± SEM. Two-tailed Student's t-tests were employed for statistical evaluation. Source data are provided as a Source Data file.

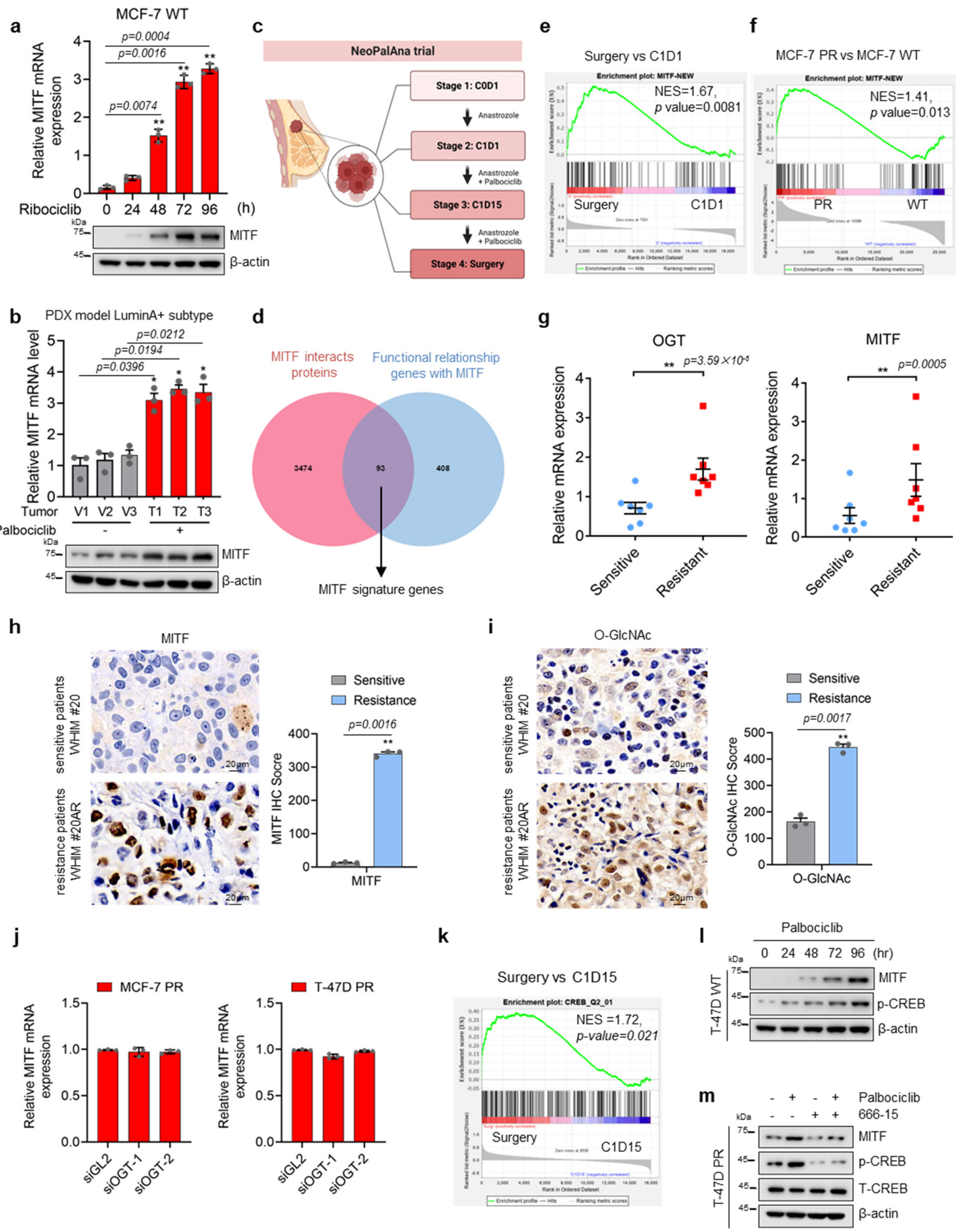

a
MCF-7 WT
Relative MITF mRNA expression
p=0.0004
p=0.0016
p=0.0074
Ribociclib 0 24 48 72 96 (h)
MITF
β-actin
b
PDX model LuminA+ subtype
Relative MITF mRNA level
p=0.0212
p=0.0194
p=0.0396
Tumor V1 V2 V3 T1 T2 T3
Palbociclib - +
MITF
β-actin
c
NeoPalAna trial
Stage 1: C0D1
Anastrozole
Stage 2: C1D1
Anastrozole + Palbociclib
Stage 3: C1D15
Anastrozole + Palbociclib
Stage 4: Surgery
d
MITF interacts proteins
Functional relationship genes with MITF
3474
93
408
MITF signature genes
e
Surgery vs C1D1
Enrichment plot: MITF-NEW
NES=1.67, p value=0.0081
Surgery
C1D1
f
MCF-7 PR vs MCF-7 WT
Enrichment plot: MITF-NEW
NES=1.41, p value=0.013
PR
WT
g
OGT
p=3.59×10⁻⁶
MITF
p=0.0005
Relative mRNA expression
Sensitive
Resistant
h
MITF
sensitive patients WHIM #20
resistance patients WHIM #20AR
Sensitive
Resistance
p=0.0016
MITF IHC Socre
MITF
i
O-GlcNAc
sensitive patients WHIM #20
resistance patients WHIM #20AR
Sensitive
Resistance
p=0.0017
O-GlcNAc IHC Socre
O-GlcNAc
j
MCF-7 PR
T-47D PR
Relative MITF mRNA expression
siGL2
siOGT-1
siOGT-2
k
Surgery vs C1D15
Enrichment plot: CREB_Q2_01
NES =1.72, p-value=0.021
Surgery
C1D15
l
Palbociclib
0 24 48 72 96 (hr)
T-47D WT
MITF
p-CREB
β-actin
m
Palbociclib
666-15
T-47D PR
MITF
p-CREB
T-CREB
β-actin

Supplementary Figure 6. MITF is activated in response to palbociclib and elevated in tumors from palbociclib-resistant breast cancer patients

(**a**) MCF-7 cells were collected after indicated treatments and then subjected to qPCR or immunoblotting to examine the expression of indicated genes (n = 3 independent experiments) and proteins. (**b**) PDX model LuminA+ tumors from mice were collected after indicated treatments and then subjected to qPCR and immunoblotting to examine the expression of indicated genes (n = 3 independent experiments) and proteins. (**c**) Schematic of the stages of the NeoPalAna trial. (**d**) Venn diagram to show the overlapping of proteins that interact with MITF (by mass-spec analysis) and genes that are functionally associated with MITF (by PahtwayNet analysis). A total of 93 genes were identified and these genes were defined as MITF signature geneset. (**e-f**) GSEA profiling to show enrichment of the MITF signature geneset in the Surgery group vs. C1D1 group (**e**), and MCF-7 PR cells vs. MCF-7 cells (**f**). (**g**) Palbociclib-resistant and sensitive breast cancer PDX cells were collected and followed by qPCR to examine the expression of indicated genes (n = 3 independent experiments). (**h-i**) Representative images of IHC staining and quantification results for MITF (**h**) and O-GlcNAc (**i**) in PDX lines WHIM 20AR and WHIM 20 (n = 3 independent experiments). The scale bar represents 20 μm. (**j**) MCF-7 PR and T-47D PR cells were collected after treatments as indicated and then subjected to qPCR to examine the expression of genes as indicated (n = 3 independent experiments). (**k**) GSEA profiling to show enrichment of the CREB geneset in the Surgery group vs. C1D15 group. (**l-m**) T-47D WT cells (**l**) and T-47D PR cells (**m**) were collected after indicated treatments and then followed by immunoblotting for the indicated proteins. (n = 3 independent experiments). **, $p \leq 0.01$. All error bars are expressed as mean ± SEM. Two-tailed Student's t-tests were employed for statistical evaluation. Source data are provided as a Source Data file.
