# Supplementary material for: O-GlcNAcylation of MITF regulates its activity and CDK4/6 inhibitor resistance in breast cancer
Source: Nat Commun. 2024 Jul 3;15:5597. doi: 10.1038/s41467-024-49875-w (PMC11222436; doi:10.1038/s41467-024-49875-w)
Supplement: Supplementary file 2 — Reporting Summary [file 41467_2024_49875_MOESM2_ESM.pdf]

nature portfolio

Corresponding author(s): Wenge Zhu

Last updated by author(s): Jun 17, 2024

Reporting Summary

Nature Portfolio wishes to improve the reproducibility of the work that we publish. This form provides structure for consistency and transparency in reporting. For further information on Nature Portfolio policies, see our Editorial Policies and the Editorial Policy Checklist.

Statistics

For all statistical analyses, confirm that the following items are present in the figure legend, table legend, main text, or Methods section.

n/a	Confirmed	
[ ]	[x]	The exact sample size (*n*) for each experimental group/condition, given as a discrete number and unit of measurement
[ ]	[x]	A statement on whether measurements were taken from distinct samples or whether the same sample was measured repeatedly
[ ]	[x]	The statistical test(s) used AND whether they are one- or two-sided<br>*Only common tests should be described solely by name; describe more complex techniques in the Methods section.*
[x]	[ ]	A description of all covariates tested
[x]	[ ]	A description of any assumptions or corrections, such as tests of normality and adjustment for multiple comparisons
[ ]	[x]	A full description of the statistical parameters including central tendency (e.g. means) or other basic estimates (e.g. regression coefficient) AND variation (e.g. standard deviation) or associated estimates of uncertainty (e.g. confidence intervals)
[ ]	[x]	For null hypothesis testing, the test statistic (e.g. *F*, *t*, *r*) with confidence intervals, effect sizes, degrees of freedom and *P* value noted<br>*Give P values as exact values whenever suitable.*
[x]	[ ]	For Bayesian analysis, information on the choice of priors and Markov chain Monte Carlo settings
[x]	[ ]	For hierarchical and complex designs, identification of the appropriate level for tests and full reporting of outcomes
[x]	[ ]	Estimates of effect sizes (e.g. Cohen's *d*, Pearson's *r*), indicating how they were calculated

Our web collection on statistics for biologists contains articles on many of the points above.

Software and code

Policy information about availability of computer code

Data collection	Western Blot: Li-cor imager<br>qPCR: Bio-Rad CFX96 device<br>Immunofluorescence: Evos m5000 imaging microscope<br>Plate reader: SpectraMax iD3 Multi-Mode Microplate Readers<br>MS:Thermo Scientific Easy nLC-1000 system coupled with Orbitrap Fusion Lumas Tribrid Mass Spectrometer
Data analysis	Statistical analysis: Graph Pad Prism 9.0 version<br>Western blot analysis: Image Studio Lite Ver 5.2

For manuscripts utilizing custom algorithms or software that are central to the research but not yet described in published literature, software must be made available to editors and reviewers. We strongly encourage code deposition in a community repository (e.g. GitHub). See the Nature Portfolio guidelines for submitting code & software for further information.

Data

Policy information about availability of data

All manuscripts must include a data availability statement. This statement should provide the following information, where applicable:

- Accession codes, unique identifiers, or web links for publicly available datasets
- A description of any restrictions on data availability
- For clinical datasets or third party data, please ensure that the statement adheres to our policy

Raw RNA-seq data and ChIP-seq data have been deposited in the Gene Expression Omnibus (GEO) database and are associated with the accession number GSE 234514 (https://www.ncbi.nlm.nih.gov/geo/query/acc.cgi?acc=GSE234514) and GSE 234515 (https://www.ncbi.nlm.nih.gov/geo/query/acc.cgi?acc=GSE234515). The DNA microarray data of the NeoPalAna dataset is associated with the accession number GSE93204 (https://www.ncbi.nlm.nih.gov/geo/query/acc.cgi?acc=GSE93204). Mass spectrometry data have been deposited in the ProteomeXchange via the PRIDE partner repository and are associated with the accession number PXD042763 (https://www.ebi.ac.uk/pride/archive/projects/PXD042763) and PXD042838 (https://www.ebi.ac.uk/pride/archive/projects/PXD042838).
All remaining data can be found in the Article, Supplementary, and Source Data files. Source data are provided in this paper.
Any further data or details needed about the paper can be obtained from the corresponding author upon request.

Research involving human participants, their data, or biological material

Policy information about studies with human participants or human data. See also policy information about sex, gender (identity/presentation), and sexual orientation and race, ethnicity and racism.

Reporting on sex and gender	N/A
Reporting on race, ethnicity, or other socially relevant groupings	N/A
Population characteristics	N/A
Recruitment	N/A
Ethics oversight	N/A

Note that full information on the approval of the study protocol must also be provided in the manuscript.

Field-specific reporting

Please select the one below that is the best fit for your research. If you are not sure, read the appropriate sections before making your selection.

[x] Life sciences   [ ] Behavioural & social sciences   [ ] Ecological, evolutionary & environmental sciences

For a reference copy of the document with all sections, see nature.com/documents/nr-reporting-summary-flat.pdf

Life sciences study design

All studies must disclose on these points even when the disclosure is negative.

Sample size	No sample size calculation was performed. The sample size was determined considering the variations and mean values of the samples, or based on previous observations or a standard protocol in the field. Sample size for each experiment is indicated in the figures or corresponding figure legends, or in the "Statistics and reproducibility" part in the manuscript.
Data exclusions	No data was excluded from analyses.
Replication	All biological replicates are obtained from biologically independent experiments. All attempts at replication were successful. The experiment numbers are indicated in the figure legends or "Statistics and reproducibility" part.
Randomization	Samples/mice/participants were randomized allocated into control and experimental groups.
Blinding	The investigators were blinded to group allocation during data collection.

Reporting for specific materials, systems and methods

We require information from authors about some types of materials, experimental systems and methods used in many studies. Here, indicate whether each material, system or method listed is relevant to your study. If you are not sure if a list item applies to your research, read the appropriate section before selecting a response.

Materials & experimental systems

n/a	Involved in the study	
☐	☒	Antibodies
☐	☒	Eukaryotic cell lines
☒	☐	Palaeontology and archaeology
☐	☒	Animals and other organisms
☒	☐	Clinical data
☒	☐	Dual use research of concern
☒	☐	Plants

Methods

n/a	Involved in the study	
☐	☒	ChIP-seq
☒	☐	Flow cytometry
☒	☐	MRI-based neuroimaging

Antibodies

Antibodies used

Anti-MITF (CST, Cat#12590; 1:1000);
Anti-phospho-Rb (Ser 780) (CST #9307; 1:1000);
Anti-phospho-Rb (Ser 807/811) (CST #8516; 1:1000);
Anti-Total-Rb (CST #9309; 1:1000);
Anti-p21 (CST #2947; 1:1000);
Anti-β-Actin (CST #3700; 1:1000);
Anti-PARP (CST #9532; 1:1000);
Anti-OGT (CST #24083; 1:1000);
Anti-His-tag (CST #12698; 1:1000);
Anti-FLAG-tag (CST #14793; 1:1000);
Anti-Myc-tag (CST #2276; 1:1000);
Anti-HA-tag (CST #3724; 1:1000);
Anti-14-3-3 (pan) (CST #95422; 1:1000);
Anti-Lamin B1 (CST #13435; 1:1000);
Anti-phospho-CREB (Ser 133) (CST #9198; 1:1000);
Anti-total-CREB (CST #9197; 1:1000);
Anti-O-GlcNAc (RL2) (Abcam #ab2739; 1:1000)

Validation

All the antibodies listed above were validated in western blotting.
All commercial antibodies have been validated by the manufacturers.

Eukaryotic cell lines

Policy information about cell lines and Sex and Gender in Research

Cell line source(s)

MCF-7 (ATCC, Cat #HTB-22)
T-47D (ATCC, Cat #HTB-133)

Authentication

All cell lines were not authenticated beyond inspection based on morphological criteria

Mycoplasma contamination

The cell lines are routinely tested using mycoplasma detection kit. All cell lines were tested negative for mycoplasma.

Commonly misidentified lines
(See ICLAC register)

No commonly misidentified cell lines were used.

Animals and other research organisms

Policy information about studies involving animals; ARRIVE guidelines recommended for reporting animal research, and Sex and Gender in Research

Laboratory animals

Nude mice (6-8 weeks) Strain #002019

Wild animals

This study did not involve wild animals

Reporting on sex

Female nude mice were employed

Field-collected samples

This study did not involve field-collected samples

Ethics oversight

Animal procedures were performed according to a protocol approved by the Institutional Animal Care and Use Committee (IACUC) at George Washington University

Note that full information on the approval of the study protocol must also be provided in the manuscript.

Plants

Seed stocks: N/A

Novel plant genotypes: N/A

Authentication: N/A

ChIP-seq

Data deposition

- [x] Confirm that both raw and final processed data have been deposited in a public database such as GEO.
- [x] Confirm that you have deposited or provided access to graph files (e.g. BED files) for the called peaks.

Data access links
May remain private before publication.
GSE 234515

Files in database submission
GSM7469924 MCF-7 WT, MITF, ChIP 1
GSM7469925 MCF-7 WT, MITF, ChIP 2
GSM7469926 MCF-7 WT, MITF, ChIP 3
GSM7469927 MCF-7 PR, MITF, ChIP 1
GSM7469928 MCF-7 PR, MITF, ChIP 2
GSM7469929 MCF-7 PR, MITF, ChIP 3

Genome browser session
(e.g. UCSC)
USCS

Methodology

Replicates
MCF-7 WT MITF (3 replicates)
MCF-7 PR MITF (3 replicates)

Sequencing depth: Each experiment >20M reads, single end 50, single end 75, single end 100, paired end 100.

Antibodies: MITF

Peak calling parameters: All settings described on ENCODE portal.

Data quality: All validation and QC are described on the ENCODE portal.

Software: IGV_2.13.0
