# Supplementary material for: O-GlcNAcylation of MITF regulates its activity and CDK4/6 inhibitor resistance in breast cancer
Source: Nat Commun. 2024 Jul 3;15:5597. doi: 10.1038/s41467-024-49875-w (PMC11222436; doi:10.1038/s41467-024-49875-w)
Supplement: Supplementary file 3 — Description of Additional Supplementary Files [file 41467_2024_49875_MOESM3_ESM.pdf]

Legends of the Supplementary data

1. Supplementary Data1

This file contains the QPCR Primers and RT-PCR Primers

2. Supplementary Data2

This file contains the 2 RNA seq data

(1) MCF-7 PR & MCF-7 WT

(2) MCF-7 PR & MCF-7 PR-shMITF

3. Supplementary Data3

This file contains the Mass spec data of MITF interact proteins and the MITF signature gene sets
